# Supplementary material for: Epidemiology of patients treated for multiple myeloma using a new algorithm in the French national health insurance database (SNDS): Results from the MYLORD study
Source: PLoS One. 2025 May 8;20(5):e0322474. doi: 10.1371/journal.pone.0322474 (PMC12061088; doi:10.1371/journal.pone.0322474)
Supplement: S1 File — Supporting information for the article detailed MYLORD algorithm (S1 Appendix), the study flowchart (S1 Fig), the description of Patient characteristics at inclusion in the MYLORD (S1 Table), prevalence rates of multiple myeloma in France from 2014 to 2020 based on MYLORD study population (S2 Table), extrapolated number of prevalent MM patients in France from 2014 to 2020 (S3 Table), incidence rates of multiple myeloma in France from 2014 to 2020 based on MYLORD study population (S4 Table) and extrapolated number of incident MM patients in France from 2014 to 2020 (S5 Table). (DOCX) [file pone.0322474.s001.docx]

**Supplementary materials**

S1 Appendix. MYLORD algorithm

All adults (≥18 years) with at least one hospital stay for MM (PD, RD or SAD with ICD-10 C90*)

**and/or** with LTD status for MM (ICD-10 C90*)

**and** treated by MM specific drugs or chemotherapy or Autologous Stem Cell Transplantation (ASCT) from January 1^st^, 2006 to December 31^st^, 2020 were included.

**MM specific drugs with ATC codes**

| L01AA03, Melphalan |
| --- |
| H02AB07, Prednisone |
| L04AX02, Thalidomide |
| L01XX32, Bortezomib |
| L04AX04, Lenalidomide |
| H02AB02, Dexamethasone |
| L01CA02, Vincristine |
| L01DB01, Adriamycin/doxorubicin |
| L01XC24, Daratumumab |
| L01AA01, Cyclophosphamide |
| L01XX42, Panobinostat |
| L01XX50, Ixazomib |
| L01XX45, Carfilzomib |
| L01AA09, Bendamustine |
| L04AX06, Pomalidomide |
| L01XX52, Venetoclax |
| L03AX16, Plerixafor |
| H02AB04, Methylprednisolone |
| L01XC38, Isatuximab |

**Chemotherapy and autologous stem cell transplant (ASCT)**

The following ICD-10 codes were used for the identification of patients:

- Z51.1 “Chemotherapy session for tumor” in PMSI-MCO (in medicine, surgery, obstetric units)
- code 05 “Chemotherapy” in PMSI-HAD (in at home hospitalization)

For transplantation, the GHM 27Z03Z "ASCT" was used.

In addition, all adults treated with lenalidomide or thalidomide (the same drug dispensed at least twice within 90 days) AND with at least 2 Serum Protein Electrophoresis (SPEP) or Urine Protein Electrophoresis (UPEP) within 4 months of the year after first dispensing of the drug **and** without hospital records on the database for indications other than MM were included.

**Hospital stays for patients with a diagnosis other than MM**:

- myelodysplastic syndrome (PD, RD or SAD with ICD-10 D46*)
- and/or follicular lymphoma (PD, RD or SAD with ICD-10 C82*)
- and/or diffuse non-Hodgkin lymphoma (PD, RD or SAD with ICD-10 C83*)
- and/or peripheral and cutaneous T cell lymphoma (PD, RD or SAD with ICD-10 C84*)
- and/or other non-Hodgkin lymphoma (PD, RD or SAD with ICD-10 C85*)
- and/or osteomyelofibrosis (PD, RD or SAD with ICD-10 C47.4)
- and/or acute panmyelosis with myelofibrosis (PD, RD or SAD with ICD-10 C94.4)
- and/or POEMS syndrome (PD, RD or SAD with ICD-10 D47.7)
- and/or amyloidosis (PD, RD or SAD with ICD-10 E85*)

S1 Fig. Flow chart


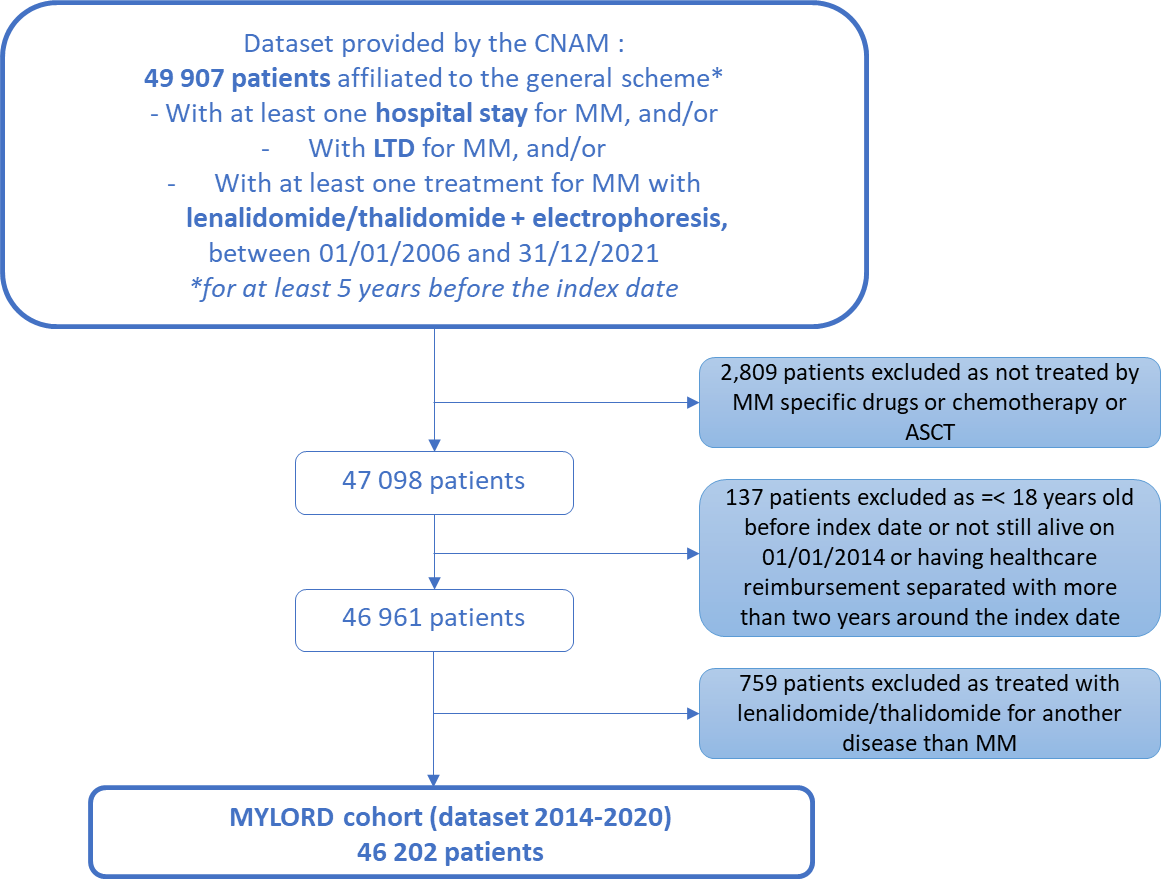


S1 Table Patient characteristics at inclusion in the MYLORD study (n=46,202)- Patients affiliated to the health insurance’s general scheme

| Parameters | Statistics | 2014  N=18,659 | 2015  N=20,366 | 2016  N=22,020 | 2017  N=23,791 | 2018  N=25,660 | 2019  N=27,352 | 2020  N=28,962 |
| --- | --- | --- | --- | --- | --- | --- | --- | --- |
| Age (in years) | Mean (±SD) | 69.01 (+/-12.97) | 69.11 (+/-13.02) | 69.24 (+/-13.06) | 69.36 (+/-13.06) | 69.54 (+/-12.99) | 69.76 (+/-12.90) | 69.92 (+/-12.83) |
|  | Median | 70.00 | 70.00 | 70.00 | 70.00 | 71.00 | 71.00 | 71.00 |
|  | Q1 ; Q3 | 62.00 ; 79.00 | 62.00 ; 79.00 | 62.00 ; 79.00 | 62.00 ; 79.00 | 62.00 ; 79.00 | 62.00 ; 79.00 | 62.00 ; 79.00 |
| Prevalence by age group (in patients) | 18-64 yo | 6,086 | 6,535 | 7,02 | 7,453 | 7,858 | 8,172 | 8,485 |
|  | 65-69 yo | 2,894 | 3,220 | 3,436 | 3,614 | 3,837 | 3,938 | 4,080 |
|  | 70-74 yo | 2,597 | 2,86 | 3,264 | 3,746 | 4,161 | 4,764 | 5,277 |
|  | 75-79 yo | 2,809 | 3,039 | 3,051 | 3,277 | 3,598 | 3,812 | 4,059 |
|  | ≥ 80 yo | 4,273 | 4,712 | 5,249 | 5,701 | 6,206 | 6,666 | 7,061 |
| Sex | Men | 9,098 (48.76%) | 9,931 (48.76%) | 10,707 (48.62%) | 11,645 (48.95%) | 12,736 (49.63%) | 13,579 (49.65%) | 13,310 (49.70%) |
|  | Women | 9,561 (51.24%) | 10,435 (51.24%) | 11,313 (51.38%) | 12,146 (51.05%) | 12,924 (50.37%) | 13,773 (50.35%) | 13,471 (50.30%) |
| LTD for MM |  | 11,835 (63.4%) | 12,727 (62.5%) | 13,669 (62.1%) | 14,743 (62.0%) | 15,951 (62.2%) | 16,788 (61.4%) | 17,559 (60.6%) |

S2 Table Prevalence rates of multiple myeloma in France from 2014 to 2020 based on MYLORD study population (n=46,202)- Patients affiliated to the health insurance’s general scheme

| **Year** | **# prevalent patients*** | **# general regimen patients** | **Crude rates for 100,000 PY** | **Standardized rates for 100,000 PY** |
| --- | --- | --- | --- | --- |
| 2014 | 18,659 | 52,305,223 | 35.67 (35.17-36.19) | 17.50 (17.23-17.78) |
| 2015 | 20,366 | 52,832,903 | 38.55 (38.02-39.08) | 18.68 (18.39-18.96) |
| 2016 | 22,020 | 52,974,762 | 41.57 (41.02-42.12) | 19.98 (19.69-20.27) |
| 2017 | 23,791 | 53,652,057 | 44.34 (43.78-44.91) | 21.00 (20.70-21.30) |
| 2018 | 25,660 | 54,372,658 | 47.19 (46.62-47.77) | 21.98 (21.68-22.28) |
| 2019 | 27,352 | 55,658,786 | 49.14 (48.56-49.73) | 22.39 (22.09-22.69) |
| 2020 | 28,962 | 59,156,067 | 48.96 (48.40-49.53) | 22.34 (22.06-22.63) |

*Numbers not extrapolated to France

PY=Person-Years

The crude rates were calculated using the number of patients recorded in the general regimen (source: CNAM)

The standardized rates were calculated using the age distribution in the world population (Doll et al. 1966)

S3 Table Extrapolated number of prevalent MM patients in France* from 2014 to 2020

| **Year** | **Total** | **Men** | **Women** | **18 - 64**  **years** | **65 - 69 years** | **70 - 74 years** | **75 - 79 years** | **>80 years** |
| --- | --- | --- | --- | --- | --- | --- | --- | --- |
| 2014 | 23,673 | 11,553 | 12,120 | 7,796 | 3,645 | 3,380 | 3,735 | 5,117 |
| 2015 | 25,691 | 12,539 | 13,152 | 8,309 | 4,048 | 3,674 | 4,031 | 5,629 |
| 2016 | 28,266 | 13,847 | 14,419 | 8,851 | 4,579 | 3,973 | 4,192 | 6,671 |
| 2017 | 30,264 | 14,926 | 15,338 | 9,306 | 4,800 | 4,610 | 4,370 | 7,178 |
| 2018 | 32,347 | 16,165 | 16,182 | 9,682 | 5,078 | 5,163 | 4,698 | 7,726 |
| 2019 | 33,534 | 16,704 | 16,830 | 9,877 | 5,035 | 5,784 | 4,811 | 8,027 |
| 2020 | 33,675 | 16,844 | 16,831 | 9,852 | 4,971 | 6,504 | 5,007 | 7,341 |

*Metropolitan and overseas France

S4 Table Incidence rates of multiple myeloma in France from 2014 to 2020 based on MYLORD study population (n=46,202)- Patients affiliated to the health insurance’s general scheme

| **Year** | **# incident patients*** | **# general regimen patients** | **Crude rates for 100,000 PY** | **Standardized rates for 100,000 PY** |
| --- | --- | --- | --- | --- |
| 2014 | 4,125 | 52,305,223 | 7.89 (7.65-8.13) | 3.80 (3.67-3.93) |
| 2015 | 4,167 | 52,832,903 | 7.89 (7.65-8.13) | 3.80 (3.67-3.93) |
| 2016 | 4,382 | 52,974,762 | 8.27 (8.03-8.52) | 3.92 (3.79-4.05) |
| 2017 | 4,678 | 53,652,057 | 8.72 (8.47-8.97) | 4.07 (3.94-4.20) |
| 2018 | 4,736 | 54,372,658 | 8.71 (8.47-8.96) | 3.99 (3.86-4.12) |
| 2019 | 4,755 | 55,658,786 | 8.54 (8.30-8.79) | 3.80 (3.68-3.92) |
| 2020 | 4,825 | 59,156,067 | 8.16 (7.93-8.39) | 3.67 (3.55-3.78) |

*Numbers not extrapolated to France

PY=Person-Years

The crude rates were calculated using the number of patients recorded in the general regimen (source: CNAM)

The standardized rates were calculated using the age distribution in the world population (Doll et al. 1966)

S5 Table Extrapolated number of incident MM patients in France* from 2014 to 2020

| **Year** | **Total** | **Men** | **Women** | **18-64 years** | **65 - 69 years** | **70 - 74 years** | **75 - 79 years** | **>80 years** |
| --- | --- | --- | --- | --- | --- | --- | --- | --- |
| 2014 | 5,223 | 2,592 | 2,631 | 1,671 | 791 | 721 | 836 | 1,204 |
| 2015 | 5,259 | 2,646 | 2,613 | 1,686 | 771 | 754 | 849 | 1,199 |
| 2016 | 5,634 | 2,817 | 2,817 | 1,739 | 889 | 772 | 790 | 1,444 |
| 2017 | 5,955 | 3,106 | 2,849 | 1,793 | 866 | 916 | 916 | 1,464 |
| 2018 | 5,979 | 3,224 | 2,755 | 1,698 | 945 | 946 | 916 | 1,474 |
| 2019 | 5,838 | 3,034 | 2,804 | 1,615 | 836 | 1,048 | 885 | 1,454 |
| 2020 | 5,608 | 2,839 | 2,769 | 1,574 | 836 | 1,084 | 839 | 1,275 |

*Metropolitan and overseas France
